# What impact could the legalisation of recreational cannabis have on the health of the United Kingdom? Lessons from the rest of the world

**DOI:** 10.1192/bjp.2024.4

**Published:** 2024-04

**Authors:** Emmert Roberts

**Affiliations:** Addiction Psychiatry, National Addiction Centre, Institute of Psychiatry, Psychology and Neuroscience, King’s College London and the South London and the Maudsley NHS Foundation Trust

## Abstract

Several jurisdictions across the globe have introduced legislation to legally permit the sale and consumption of recreational cannabis. This editorial considers current evidence from the rest of the world and asks how this might inform the possible consequences of *“legalisation”* models in the United Kingdom.

## Editorial

Over the last ten years several jurisdictions worldwide have legalised the regulated sale and recreational use of cannabis products. Widely differing models of legalisation have been enacted across the globe ranging from more tightly regulated and government-controlled markets (e.g., in several Canadian provinces) to full for-profit commercial sale and supply (e.g., some states in the US). Although the United Kingdom (UK) has thus far only relaxed legislation to permit medical prescription, increasing calls from advocates and changing public opinion are likely to result in serious consideration of the potential consequences of any proposed legalisation policy.

## Potential benefits

Irrespective of the regulatory controls or retail strategies enacted by each jurisdiction every global example of legalisation has made cannabis products more available, more affordable, and more easily advertised to potential consumers. (1, 2) Potential benefits include substantial taxable revenue generation and specific reductions in cannabis-related crimes that have historically been used to overpolice minorities and communities of colour. (2) Arguments have also been made regarding the potential for legalisation to reduce stigma directed against people who use cannabis products and to address any current barriers people may encounter when seeking appropriate treatment or harm reduction measures for problematic cannabis use. These benefits however must be balanced against any potential adverse public health considerations mediated via post-legalisation changes in individual and population level consumption patterns.

## Potential risks

Notwithstanding the benefits of product quality assurance that occur when moving to an unadulterated and regulated market almost all jurisdictions report a post-legalisation increase in the population level of cannabis consumption and, by extension, increased rates of cannabis addiction. (3) Many areas also observe significantly increased cannabis product potency, the persistence of unregulated or ‘black’ markets despite legalisation and substantial diversification in the types of cannabis products available via different routes of administration. (2) Demand upon the UK National Health Service (NHS) is currently at an all-time high and there is mounting evidence of post legalisation increases in cannabis-related emergency department visits in peer countries. (1, 2, 4) Global reports also suggest that several cannabis-related mental and physical health harms may increase upon legalisation with these likely to be concentrated among teenagers, young adults, pregnant women and those with existing mental health disorders. (5) Increased availability following legalisation has been linked to increased rates of psychosis and cannabis-involved pregnancies, (4, 6) with this association more pronounced among people who have been less exposed to cannabis products prior to legalisation. Given cannabis use is a significant risk factor for people, with and without underlying psychosis, to transition to schizophrenia spectrum disorders any significant post legalisation increase in availability, potency or consumption may have a substantial impact on population level mental health. (7) Depending on the legalisation model adopted there are also reports of significant increases in cannabis-related road traffic accidents and spikes in accidental child poisonings. (2, 8, 9)

If the UK policy or ‘Overton’ window opens wide enough for cannabis product legalisation to become a viable policy option, the additional pressures likely placed on the UK health and social care system, and in particular the mental health and addiction treatment systems, may further stretch the currently limited resources in these settings and exacerbate identified underlying issues including chronic underfunding and lack of an adequately trained workforce.

### “Legalisation”

The term “legalisation” is neither binary nor refers to a singular policy decision. Different models of legalisation, with different decisions relating to factors such as commercialisation and risk mitigation, are thus likely to result in very different scenarios with respect to health outcomes.(2) As such there is likely to be a theoretical statute by which recreational cannabis legalisation in the UK, on aggregate, reduces the overall level of societal harm it inflicts compared to the current legal framework. However, this hypothetical legislation would almost certainly require extremely strict regulatory sales practices and hypothecated funding from generated revenue to combat any increase in cannabis-related harms. (1, 2) Future shifts in political power, alongside innovative practices of industries that legally produce and market addictive products, mean any new legislation is susceptible to commercial or governmental attempts at deregulation and market expansion. The requirement of a guarantee that these laws and any public health protections would not be ‘watered down’ would appear in tension with the concept of UK parliamentary sovereignty. Given the extreme unlikelihood of wholesale repeal of a legalisation statute - there are currently no international examples of ‘re-banning’ similar products or indeed of significantly tightening regulatory controls once the decision has been made to legalise - this should factor into considerations of potential longer-term harm to the nation’s health. As both benefits and harms disproportionately affect people in society who typically experience minoritsation, equitable consideration needs to be given to these groups, particularly as the political landscape, corporate influence, product innovation and advocate priorities change over time.

Stating a blanket position as a ‘yes or no’ to cannabis product “legalisation” fails to consider the nuance of a multitude of policy decisions that would impact the consequences of any proposed legislation. As such balancing the risks and benefits is necessarily a complex process. Further, decisions relating to commercialisation, proposed mitigations against potential harms and the ability to amend any provisions after statute passage all alter the risk-benefit equation of such a decision. As further international evidence emerges relating to the short-, medium- and longer-term consequences of legalisation across other jurisdictions some of these issues may become clearer. At the current moment in time, a substantial number of policy questions remain unanswered and pressure on the UK mental health and addiction treatment systems is at an all-time high. This limits the current UK government’s ability to draft a piece of future proofed legislation that would definitively result in the sustained protection of the nation’s health in an era of legally sanctioned supply and consumption.

## Data Availability

N/A
